# Co-designing eCap-CoDe: A mobile health application for primary health care-based dementia care in rural Uganda

**DOI:** 10.1371/journal.pdig.0001389

**Published:** 2026-04-30

**Authors:** Edith K. Wakida, Christine K. Karungi, William Wasswa, Recho Katusabe Ajok, Godfrey Z. Rukundo, Ou Zhang, Alexandra Lopez-Vera, Zohray M. Talib, Celestino Obua

**Affiliations:** 1 Department of Medical Education, California University of Science and Medicine, Colton, California, United States of America; 2 Research and Development Unit, Alpha Center for Research Administration, Mbarara, Uganda; 3 Faculty of Applied Science and Technology, Mbarara University of Science and Technology, Mbarara, Uganda; 4 Department of Psychiatry, Mbarara University of Science and Technology, Mbarara, Uganda; 5 Department of Psychiatry and Behavioral Neurosciences, McMaster University, Hamilton, Ontario, Canada; Jos University Teaching Hospital, NIGERIA

## Abstract

Dementia is an emerging public health challenge in low- and middle-income countries (LMICs), yet it remains underdiagnosed in rural Uganda, where primary health care (PHC) providers often lack tools, training, and data systems for early detection and management. Mobile health (mHealth) applications can enhance provider capacity, improve data capture, and strengthen feedback systems. This study explored the perspectives of PHC providers and District Health Teams (DHTs) to inform the co-design of eCap-CoDe, a mobile application for community-based dementia care in rural Uganda. We conducted in-depth interviews with 31 participants from two rural districts: 23 PHC providers (medical/clinical officers and nurses) and 8 DHT members. Participants were purposively sampled for diversity in cadre, experience, and facility type. Data were thematically analyzed using the Consolidated Framework for Implementation Research (CFIR), with four *a priori* domains, i.e., content, user experience, organizational, and service delivery, guiding coding and analysis. *Content requirements* - included dementia-specific screening and management tools, modular in-app training aligned with the WHO mhGAP Intervention Guide, and structured data capture integrated with Uganda’s Health Management Information System (HMIS). *User experience needs:* emphasized simple, intuitive interfaces with dropdown menus, checkboxes, audio-visual decision support, and offline functionality to address connectivity gaps. *Organizational requirements:* prioritized interoperability with District Health Information System 2 (DHIS2), integration with supervisory workflows, and dementia-specific performance indicators. *Service delivery* needs: focused on real-time feedback loops, reducing duplicate documentation, and potential expansion to other common conditions to enhance utility and uptake. Co-designing mHealth tools with end-users ensures alignment with the realities of workflows, systems, and infrastructure. eCap-CoDe addresses capacity, data, and feedback gaps in rural dementia care and offers a scalable model for integrating digital tools into PHC in Uganda and similar LMICs. Pilot testing will assess the feasibility, usability, and impact before scaling up.

## Author summary

In rural Uganda, dementia often goes undetected because primary health care workers lack training, diagnostic tools, and clear reporting systems. We worked directly with health workers and district supervisors to design eCap-CoDe, a mobile application that provides in-app training, standardized dementia screening, and real-time feedback on cases. This collaborative process ensures the tool fits existing workflows and national reporting systems, making it easier for health workers to identify and manage dementia cases. eCap-CoDe could serve as a model for integrating similar digital tools for other health conditions in low-resource settings.

## Introduction

Dementia is a growing global public health concern, affecting over 57 million people worldwide, with nearly 10 million new cases each year [1,2]. More than 60% of people with dementia live in low- and middle-income countries (LMICs), where diagnostic, treatment, and care resources are limited [2,3]. In sub-Saharan Africa, including Uganda, dementia remains underdiagnosed, and available services are often fragmented, inaccessible, or unaffordable, particularly in rural areas [4,5].

In Uganda’s decentralized health system, Primary Health Care (PHC) facilities are the first point of contact for most individuals with mental and neurological conditions. However, dementia-specific policies and standardized tools for screening, diagnosis, and documentation are lacking, leaving PHC providers inadequately prepared for early detection and management [6]. District Health Teams (DHTs), responsible for supervision, depend on aggregated data that overlook community-level mental health needs [7]. These policy gaps and operational challenges limit the quality of care, hinder effective planning, and reduce accountability.

Digital health technologies, particularly mobile health (mHealth) solutions, offer opportunities to strengthen dementia care through structured data collection, decision support, and real-time feedback [8]. Yet, few such tools have been developed for or tested in rural African contexts, and even fewer are designed with direct input from frontline providers and district supervisors to ensure contextual fit. Co-designing solutions with PHC providers and DHTs is therefore essential to create interventions that are feasible, acceptable, and aligned with existing health information systems.

We are developing eCap-CoDe (electronic data capture and feedback mobile application for community-based dementia care), a multi-component mobile application designed to: (1) deliver mhGAP-informed training on dementia assessment and management [9]; (2) capture standardized patient data with online/offline functionality integrated into Uganda’s HMIS; and (3) provide automated, data-driven feedback aligned with national protocols. By addressing knowledge gaps, reducing paperwork, and improving data-informed decision-making, we hypothesize that this co-designed intervention will achieve better uptake and scalability than previous mHealth efforts [10,11].

### Overview of the eCap-CoDe mobile application

The *electronic data capture and feedback mobile application for community-based dementia care* (eCap-CoDe) is designed to strengthen dementia detection, documentation, and management in rural Uganda. It has three integrated components:

1. **Training Module:** Two self-paced modules on dementia assessment and management, informed by the WHO mhGAP-IG [9], each with pre- and post-quizzes to assess knowledge.
2. **Data Capture:** Structured entry of patient demographics, medical history, assessment, management, and follow-up, with online/offline functionality to address connectivity gaps and integration with the national Health Management Information System (HMIS).
3. **Automated Feedback:** Real-time, data-driven feedback aligned with mhGAP-IG protocols, plus a “Frequently Asked Questions” section, enabling PHC providers to improve clinical decisions and care quality.

**Fig 1:**
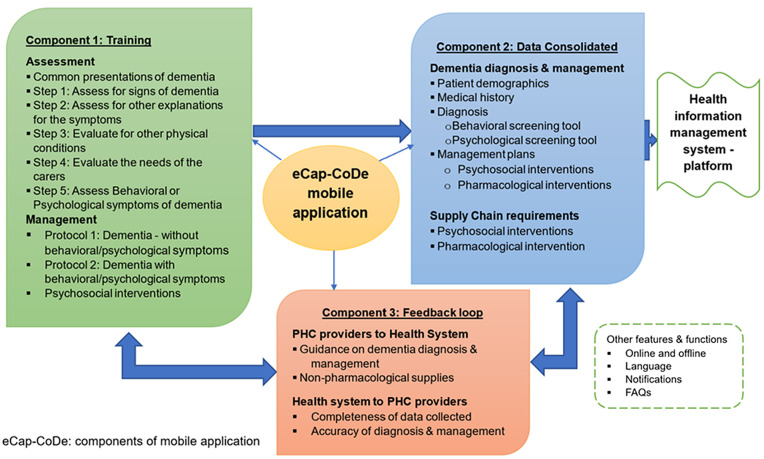
Integrated components of the eCap-CoDe mobile application, training, data capture, and real-time feedback, are designed to work together to strengthen dementia care at the primary health care level. The training module builds provider knowledge, the data capture function enables systematic recording of patient information, and the feedback mechanism delivers automated, protocol-based guidance. All components will link to the national health information system to support data-driven decision-making and improved patient outcomes.

Together, these components aim to build provider capacity, reduce documentation burden, and enable District Health Teams to access timely, actionable data for supervision and planning (Fig 1).

## Methods

### Study design and theoretical orientation

This study was a formative, qualitative inquiry using one-on-one, in-depth interviews to gather perspectives on the design of a multi-component mobile application, *eCap-CoDe* (electronic data capture and feedback mobile application for community-based dementia care), intended to support dementia care delivery in rural Uganda. A qualitative design was selected to elicit in-depth, contextually grounded insights from frontline providers and district-level health managers prior to the development of the application.

The study was informed by the Consolidated Framework for Implementation Research (CFIR) [12,13], which guided the development of interview guides, data collection, and analytic organization. CFIR was selected as the most appropriate framework for this formative study, as it supports a systematic assessment of contextual and individual factors that influence the adoption of innovations, such as mHealth tools, in low-resource settings.

For this study, we mapped:

• *Content and user experience requirements* to the CFIR domains of *individual characteristics*, *inner setting*, *process*, and *innovation characteristics*.
*• Organizational and service requirements* to the *outer setting* domain.

This framework helped structure the interview guides, the data collection tools, and later, the analytic framework [14]. Specifically, the interview guides explored perceived barriers and facilitators to dementia care and the use of digital tools; these insights were intentionally used as inputs to inform the co-design and specification of application content, user experience, organizational requirements, and service delivery requirements. Reporting followed the Consolidated Criteria for Reporting Qualitative Research (COREQ) checklist for reporting qualitative studies [15].

### Study setting

The study was conducted in two purposively selected rural districts in Uganda, referred to here as District A and District B. District A was chosen to leverage ongoing collaborations with the research team, and District B was selected for its functional hospital information management system, which could be integrated with the proposed app. The two districts are approximately 60 kilometers apart and 350 kilometers from Kampala, Uganda’s capital. Both districts are predominantly rural, with over 80% of the population residing in rural communities.

### Research team and reflexivity

The multidisciplinary research team included experts in qualitative research, implementation science, health systems research (EKW, CKK, OZ, ALV, ZMT, CO), health informatics (WW), and psychiatry with expertise in dementia care (GZR, SM). Three research assistants (VA, RKA, and PA), all with backgrounds in mental health or social sciences and prior experience in qualitative research, conducted the interviews.

Interviewers had no prior relationships. Reflexivity was addressed through team discussions both before and during data collection, aiming to minimize assumptions related to professional hierarchies and the adoption of technology. Analytic decisions were reviewed through regular team meetings to reduce individual interpretive bias.

### Participants and sampling

#### Primary Health Care (PHC) providers.

Participants were selected from four public health facilities (Health Center III and IV levels) across the two districts. Eligible participants were medical officers, clinical officers, or nursing officers directly involved in patient assessment, as stipulated by the Uganda Clinical Guidelines [16]. Based on the average staffing patterns, each health facility had 5–8 eligible providers. All eligible providers present at the time of the study were invited to participate, resulting in a total sample of 23 PHC providers (12 from District A and 11 from District B). These included two medical officers, eight clinical officers, and thirteen nursing officers. Sampling was based on provider availability rather than saturation. Although formal saturation was not used to determine sample size, iterative analysis revealed substantial thematic overlap across interviews, supporting the adequacy of the sample for the study’s formative and design-focused objectives.

#### District Health Team (DHT) members.

To understand service and organizational-level perspectives, eight DHT members were purposively selected across the two districts. These included District Health Officers (DHOs), Assistant DHOs, HMIS officers, and biostatisticians with backgrounds in medicine, nursing, public health, or health information management. Each district typically has four health management officers involved in planning and supervision. Sampling continued until information saturation was achieved, particularly among DHT participants, where all eligible individuals across both districts were included.

### Recruitment and consent procedures

Prospective participants were initially contacted by phone by the lead investigator (EKW), who explained the purpose of the study. Those who agreed were scheduled for in-person interviews and provided formal consent prior to participation. All participants were 18 years or older and provided written informed consent. Confidentiality was assured, and participants were informed that their responses would be de-identified in any publications.

### Data collection

#### Interview guides.

Semi-structured interview guides were developed, adapted, and pre-tested to ensure clarity and contextual relevance (S1 File). Guides were structured around four design domains:

1. content requirements (e.g., training needs, data capture, feedback to PHC providers),
2. user experience requirements (e.g., interface functionality, real-time feedback, data privacy, and security),
3. organizational requirements (e.g., integration with existing systems, infrastructure needs), and
4. service delivery needs (e.g., remote diagnosis, supervision, educational resources).

These domains were mapped to relevant CFIR constructs to ensure systematic exploration of implementation determinants.

PHC providers primarily addressed content and user experience domains, while DHT members concentrated on organizational and service delivery requirements, reflecting their distinct roles within the health system.

### Data collection procedures

Interviews were conducted in-person between April and May 2024 by two female research assistants (VA and PA). VA is a trained social scientist with prior experience in qualitative health research, and PA is a psychiatrist with clinical expertise in mental health and dementia care. Both interviewers received training in qualitative interviewing techniques prior to data collection. Neither interviewer had a prior relationship with study participants. Interviews took place in private spaces within health facilities or district offices to ensure confidentiality and participant comfort. All interviews were conducted in English, the official language used in Ugandan health services.

Before data collection began, the principal investigator (EKW) obtained administrative clearance from district health authorities.

- Participants were informed of the study objectives, procedures, voluntary nature of participation, and confidentiality safeguards. Written informed consent was obtained from all participants (aged 18 years and above). Verbal consent was also obtained for audio recording the interviews. Interviewers took supplementary field notes to capture contextual details and non-verbal cues.
- All interviews were conducted in quiet and private spaces within health facilities or district offices to ensure comfort and confidentiality. Interviews were conducted in English, the official language used in Ugandan health services. Each session lasted between 30 and 60 minutes, with an average duration of approximately 45 minutes.
- Participants were assigned non-identifiable codes to ensure anonymity in transcripts and publications. Data saturation was achieved, particularly among the District Health Team (DHT) members, due to the complete inclusion of all eligible individuals across the two districts.

## Data management and analysis

### Transcription and data familiarization

All audio-recorded interviews were transcribed verbatim by trained transcriptionists (PK, CA, and RAK), who were not involved in the data collection process. Transcriptions were cross-checked for accuracy through internal peer review, and EKW performed a final consistency check against the original audio files [17]. All transcripts were read and re-read by members of the research team (EKW, WW, ALV, CKK, and CO) to ensure deep familiarity with the data and to identify preliminary ideas and patterns [18].

### Analytic approach

A rapid qualitative analysis approach with purposeful data reduction was used to facilitate iterative review and interpretation of the data [19]. The coding structure was developed by EKW based on *a priori* thematic categories drawn from the CFIR-guided data collection framework and design requirements. For the PHC provider dataset, the primary themes included content and user experience requirements, while the DHT dataset focused on organizational and service requirements [14].

Data were organized using a framework matrix, where rows represented sub-themes and columns captured summarized responses, illustrative quotations, and analytic notes. This approach facilitated systematic comparison across participants and stakeholder groups.

### Coding and consensus

Coding was conducted independently by EKW, CKK, and OZ. For the DHT dataset, EKW and CKK applied a double-coding approach using a predefined codebook. Any discrepancies were discussed and resolved through consensus, with input from additional investigators (WW and GZR) [20]. Data were compared iteratively within and across participant groups to identify convergence and divergence of perspectives.

### Parallel analysis

PHC providers and DHT data were analyzed in parallel due to role-specific interview guides. PHC provider interviews focused on content and user experience requirements, whereas DHT interviews addressed organizational and service delivery needs. Given the role-specific nature of the questions, the findings were not compared for convergence or divergence; rather, each dataset was analyzed independently to generate domain-specific insights that collectively informed the multi-level design framework for the eCap-CoDe application.

### Ensuring trustworthiness

Trustworthiness was enhanced through multiple strategies. First, transcripts were produced by individuals not involved in data collection, reducing interviewer bias during transcription. Second, transcript cross-verification and double-coding increased reliability. Third, three investigators (GZR, WW, and CO) were not involved in data collection and provided critical external perspectives during data interpretation. Fourth, consensus meetings were held to finalize code groups, themes, and representative quotations. All data (transcripts, audio files, notes, and matrices) were stored securely on password-protected systems to maintain confidentiality. Participant checking (returning transcripts or findings to participants for comment or correction) was not conducted. Given the formative and design-focused nature of the study, rigor was instead ensured through investigator triangulation, double coding, consensus meetings, and iterative analytic review.

### Ethics approval and consent to participate

Ethical approval was obtained from the Mbarara University Research Ethics Committee (MUST-2024–1480), and the study was registered with the Uganda National Science and Technology (HS4064ES). Administrative clearance was obtained from the District Health Officer. All participants provided written informed consent, and privacy was ensured through anonymization and secure data storage.

## Results

We conducted in-depth interviews with 31 participants across two rural Ugandan districts: 23 PHC providers (11 females, 12 males; ages 27–59) and 8 DHT members (6 males, 2 females; ages 33–59). PHC cadres included medical officers (n = 2), clinical officers (n = 8), and nursing officers (n = 13), with varying experience levels (ranging from less than 5 to over 21 years). DHT participants came from diverse health disciplines, with most holding bachelor’s or master’s degrees and over 2 years of service. Findings were structured into four overarching domains guiding the design of the eCap-CoDe intervention:

1. Content Requirements (PHC provider perspectives)
2. User Experience Requirements (PHC provider perspectives)
3. Organizational Requirements (DHT perspectives)
4. Service Requirements (DHT perspectives)

From these domains, we identified 15 sub-themes, summarized as follows:

- *PHC Provider Sub-Themes*: 1) Training needs for dementia assessment and management, 2) Data capture processes and preferences, 3) Feedback mechanisms to support care quality, 4) Perceptions of the multi-component structure of the eCap-CoDe app, 5) Desired technical features for the app, 6) Usability and interface friendliness, 7) Workflow integration into routine PHC practice, 8) Comparative advantages over previous tools or paper-based systems
- *DHT Member Sub-Themes:* 1) Perceived value of the proposed eCap-CoDe application, 2) Organizational systems and workflows to be considered, 3) Performance monitoring indicators to embed in the app, 4) Supervision and support needs from DHT to frontline PHC workers, 5) Health Management Information System (HMIS) integration, 6) District-level support to enhance uptake and sustainability, 7) Report types and formats expected from app-generated data

These themes reveal critical multi-level design and implementation considerations for a user-centered, sustainable mobile health intervention for community dementia care.

Table 1 presents illustrative quotes aligned with each theme.

**Table 1:** Summary of Key Message with Corresponding Quotes.

Thematic Area	Key Message	Corresponding Quote
Content Requirements	Limited knowledge and tools for dementia diagnosis and management	We have a knowledge gap in diagnosing people with dementia… if you don’t make an appropriate diagnosis, sometimes even the treatment becomes problematic (Participant 1, Male PHC provider, District B)
	Cultural beliefs delay seeking care	Caretakers first think it is witchcraft… they prefer to be prayed for… older people are living alone… no one helps them (Participant 11, Male PHC provider, District A)
	Lack of specific tools beyond the Uganda Clinical Guidelines	No tool other than the Uganda Clinical Care Guidelines… we manage elderly people based on their symptoms and lab workups (Participant 9, Male PHC provider, District B)
User Experience Requirements	The app should include essential features and be user-friendly	There should be a way to retrieve and correct wrong data… each user should have a unique code… But it shouldn’t be too complex (Participant 3, Male PHC provider, District B)
	Dropdowns and textboxes are preferred for flexibility in data entry	I prefer the dropdown with a textbox for detailed information… someone may present with more than two symptoms (Participant 11, Male PHC provider, District A)
Organizational Requirements	App should support both providers and clients, and be co-designed	The app should support both providers and clients… be simple, data-free… and co-designed with all stakeholders (Participant 1, Female, DHT, District B)
	Power and network challenges must be addressed	We must plan for power and network challenges… and engage all health workers at every care point (Participant 2, Female DHT, District B)
Service Requirements	App should sync with the existing HMIS and cover all conditions	The app should sync with DHIS2 to reduce duplication and save time… Digitizing all conditions will motivate providers (Participant 1, Female, DHT, District B)
	Expect reports to match HMIS indicators	We expect the same indicators as the standard HMIS tools, captured and transmitted through the app… (Participant 3, Male, DHT, District A)

### Theme 1: Content requirement

#### Training needs for dementia assessment and management.

Participants reported significant gaps in dementia screening and management, including limited knowledge, absence of specific assessment tools, and minimal exposure to confirmed cases. Dementia is rarely the presenting complaint, often masked by other conditions, making identification difficult. Additionally, psychosocial support was the primary form of management due to a lack of medications.

*Most [patients] come with different conditions… when you probe them, that’s when you know this kind of person has dementia. We have a knowledge gap in diagnosing people with dementia. …people have been downplaying that condition because they are unaware of it. Treatment is also a problem; if you don’t make an appropriate diagnosis, sometimes even the treatment becomes problematic* ***(Participant 1, Male PHC provider, District B).***

Cultural beliefs further delayed diagnosis, with caregivers attributing symptoms to witchcraft or preferring prayers before seeking medical care. Elderly patients living alone also faced neglect.

*“Caretakers first think it is witchcraft… they prefer to be prayed for… older people are living alone… no one helps them* ***(Participant 11, Male PHC provider, District A).***

All providers stated they lacked structured dementia screening tools and relied solely on the Uganda Clinical Guidelines, which are symptom-based and do not explicitly guide cognitive or behavioral assessments.

*No tool other than the Uganda Clinical Care Guidelines… we manage elderly people based on their symptoms and lab workups* ***(Participant 9, Male PHC provider, District B).***

*We attend to them based on their presenting complaints… we have many patients and are limited by facilities* ***(Participant 6, Male PHC provider, District A).***

#### Data capture processes and preferences.

Most participants reported that dementia-related information was recorded in the patient’s treatment book, then transferred to the outpatient department (OPD) register and eventually into the DHIS2 system via HMIS Form 105. However, dementia data is often missing due to the lack of dedicated fields or tools for its documentation.

*Something is not coming out on dementia… it has been ignored… I don’t know if that diagnosis is in our register* ***(Participant 12, Female PHC provider, District B).***

*We record in their treatment book, then the OPD [outpatients department] register, then report into the DHIS 2 [district health information system]* ***(Participant 11, Male PHC provider, District A).***

Several challenges were noted, including incomplete or inaccurate data (e.g., dates, diagnoses), illegible handwriting, and inconsistencies during data transfer. Additionally, there were missed opportunities to document cases when patients left without treatment or came without their books.

*The main challenge is incomplete data, and you do not know the care that was given or what treatment to give… handwriting cannot be read well. You fidget to get what somebody entered* ***(Participant 9, Male PHC provider, District B).***

Personnel shortages and reliance on undertrained staff or volunteers contributed to data quality issues.

*We have only one data person… records are sometimes done by enrolled nurses or volunteers. It’s very easy for me to prescribe treatment, but if somebody is a volunteer and tomorrow that person is not there, it’s possible to miss information* ***(Participant 6, Male PHC provider, District A).***

#### Feedback mechanisms to support care quality.

Participants reported that current feedback mechanisms are general, not specific to dementia. Feedback typically comes from the district’s monitoring and evaluation team or health information officers and is provided quarterly, focusing on priority conditions like malaria.

*Specifically, there is no feedback on dementia… it would be interesting if there were a mechanism to give us specific feedback* ***(Participant 9, Male PHC provider, District B).***

Many noted that dementia is often overlooked in routine reviews and meetings due to low diagnosis rates and limited focus.

*We are not focusing on that… most people focus on malaria. Dementia is a rare thing on our side. I have been leaving that out unattended* ***(Participant 12, Female PHC provider, District A).***

When asked who should provide feedback on dementia care, participants mentioned various roles, including data clerks, health facility managers, and psychiatric officers. Data clerks were seen as best positioned to provide detailed updates based on system entries.

*The data clerk can notify us how many cases have been assessed and managed… he knows better because he punches it into the system* ***(Participant 6, Male PHC provider, District A).***

### Theme 2: User experience requirements

#### Perceptions of the multi-component structure of the eCap-CoDe app.

Participants expressed mixed views on the complexity of the app’s multiple components. While some found the idea of combining training, data capture, and feedback intuitive, others felt it could be challenging, especially for users with limited digital skills. Most agreed, however, that with proper training, the app would be manageable and ultimately beneficial.

*It should be designed so that when you open the app, you see training, capture… whatever you need should be stipulated… It would be complex, but I think it will still be user-friendly* ***(Participant 1, Male PHC provider, District A)***

Adaptability was viewed as essential for health workers in a digitizing world. Participants noted that initial resistance was likely but could be overcome with ongoing training and support.

*It will not be a one-day thing… after training, we need time to adapt. With stable internet access, it will be very good, with only one click, you can review all patient information* ***(Participant 9, Male PHC provider, District B).***

#### Desired technical features for the app.

Participants recommended several core features for the eCap-CoDe mobile application to support dementia assessment and care. These included clinical symptom checklists, patient demographics (age, sex, location, history, duration, diagnosis), assessment forms with appointment scheduling and reminder alerts, referral pathways, and visual tools like geo-mapping to track client origins. They emphasized the need for embedded guidelines, standard operating procedures, and demonstrations to support dementia assessment and management. Data accuracy and user accountability were also highlighted, including the ability to retrieve and correct entries and assign unique user codes.

*There should be a way to retrieve and correct wrong data. Also, each user should have a unique code so that data isn’t tampered with. Whoever enters the data should be responsible. But it shouldn’t be too complex* ***(Participant 3, Male PHC provider, District B).***

#### Usability and interface friendliness.

Participants emphasized the importance of a user-friendly design to reduce patient wait times and streamline data entry. Checkboxes were widely preferred for quick selection among multiple options.

*The checkbox will work better… You tick the correct box and make the final decision* ***(Participant 8, Female PHC provider, District B).***

For socio-demographics and presenting complaints, many favored dropdown menus paired with textboxes to allow both structured and detailed input:

*I prefer the dropdown with a textbox for detailed information… someone may present with more than two symptoms* ***(Participant 11, Male PHC provider, District A).***

There was general agreement that textboxes should be available when predefined options do not capture the needed response:

*If you cannot find a proper answer, then there should be a place where you specify it and write something that you think is fit for that question* ***(Participant 11, Male PHC provider, District B).***

#### Workflow integration into routine PHC practice.

Participants emphasized the importance of embedding dementia assessment into existing health management information systems and tools, rather than introducing a standalone system. They believed integration would enhance usability and promote adoption.

*It should not look like it is something new, but it could be integrated into our assessment tools so that it feels like part of routine* ***(Participant 8, Female PHC provider, District A).***

#### Comparative advantages over previous tools or paper-based systems.

The proposed app was seen as superior to current paper-based tools due to its potential for real-time data entry, reduced paperwork, improved reporting, and better tracking of dementia cases. It was viewed as a solution to existing gaps in data capture and management.

*It will help us generate better data… We’ll clearly know how many patients with dementia we have been missing. This gives us a simple way to visualize those cases* ***(Participant 9, Male PHC provider, District B).***

### Theme 3: Organizational requirement

#### Perceived value of the proposed eCap-CoDe application.

Participants viewed the app as a tool to improve care quality and timely reporting by syncing with the national system and ensuring complete data entry. They emphasized accessibility, suggesting the app be data-free, usable on basic phones, and include visuals/audio to support low-literacy users. They also stressed the importance of co-designing the app with input from providers, clients, the MoH, and donors.

*The app should support both providers and clients… be simple, data-free, and usable even on basic phones… and co-designed with all stakeholders* ***(Participant 1, Female, DHT, District B).***

#### Organizational systems and workflows to be considered.

Respondents highlighted infrastructure needs (smartphones, power banks, network access), alignment with clinical workflows, and the need to involve all cadres (doctors, nurses, lab staff, records officers) to ensure seamless data collection.

*We must plan for power and network challenges… and engage all health workers at every care point* ***(Participant 2, Female DHT, District B).***

#### Performance monitoring indicators to embed in the app.

Participants suggested including indicators such as data entry status, user activity, and facility-level reporting summaries. Supervisors at multiple levels should have dashboard access to monitor engagement, provide feedback, and ensure accountability.

*Supervisors need access to track usage, provide feedback, and support inactive users—access shouldn’t be limited to one person—multiple DHT members need visibility to ensure continuity, as roles rotate depending on availability* ***(Participant 2, Female, DHT, District B).***

#### Support for PHC supervision.

District teams need training, dashboard access, and integration of health assistants/educators in the supervision loop. Regular review of facility data against registers will help identify and address reporting gaps.

*Once we are registered in the system and trained, we can mentor, validate data, and solve gaps together with PHC teams* ***(Participant 3, Male, DHT, District A).***

### Theme 4: Service requirements

#### Health Management Information System (HMIS) integration.

Participants emphasized the need for seamless integration between the eCap-CoDe mobile application and the existing DHIS2 system. They recommended synchronizing the app with current paper-based HMIS tools to avoid duplication and ensure consistent reporting. Several suggested updating the HMIS to categorize dementia as a distinct condition rather than grouping it with other mental disorders. They also proposed that if digital tools are adopted, all disease conditions, not just dementia, should be digitized to encourage broader use by health workers.

*The app should sync with DHIS2 to reduce duplication and save time. If it only covers dementia, it may discourage use. Digitizing all conditions will motivate providers to use it consistently* ***(Participant 1, Female, DHT, District B).***

#### District-level support to enhance uptake and sustainability.

District Health Team (DHT) members emphasized the importance of practical training, ongoing mentorship, supportive supervision, and regular reminders to ensure PHC providers adopt and sustain use of the eCap-CoDe intervention. Joint reviews were also suggested to strengthen technical support at the facility level.

*After the training, I will mentor them, conduct CMEs [continuous medical education], and keep reminding them to use the app. Support supervision is key* ***(Participant 3, Male, DHT, District B).***

*We need hands-on training, mentoring, supervision, and joint reviews to provide comprehensive support* ***(Participant 3, Male, DHT, District A).***

#### Expected reports from ‘eCap-CoDe’ generated data.

Participants recommended that reports from the eCap-CoDe application align with existing HMIS indicators. Expected data includes the number of individuals assessed, diagnosed, and treated for dementia, disaggregated by sex and age, along with monthly outpatient/inpatient visit summaries and outcomes. Consistency between mobile app data and paper-based HMIS submissions was emphasized.

*We expect the same indicators as the standard HMIS tools, captured and transmitted through the app, and correlated with the manual reports* ***(Participant 3, Male, DHT, District A).***

## Discussion

This study identified four a priori domains comprising content, user experience, organizational, and service delivery requirements that should guide the co-design of eCap-CoDe, a mobile application to support dementia care in rural Uganda. Primary Health Care (PHC) providers emphasized the need for dementia-specific screening and management tools, integrated training, and real-time feedback. District Health Teams (DHTs) prioritized interoperability with existing systems, inclusion of dementia-specific indicators, and infrastructure adaptations to support adoption. Across both groups, integration with the national District Health Information System 2 (DHIS2), offline functionality, and streamlined workflows were viewed as essential for uptake and sustainability.

PHC providers reported limited prior training on dementia, echoing gaps documented in Nigeria [21], Kenya [22] Vietnam [23], and China [24]. In Uganda, dementia is often undetected or misattributed to cultural beliefs [25], leading to symptom-based rather than cause-based management. eCap-CoDe’s training component, informed by the WHO mhGAP-IG [26], addresses this gap through modular, self-paced learning with assessments. Decision support tools with visual and audio aids, mandatory data fields, follow-up alerts, and referral pathways mirror best practices from CommCare and CHW Central [27,28]. Integration of structured dementia data into Uganda’s Health Management Information System (HMIS) responds to long-standing limitations in paper-based reporting [29] and builds on mHealth successes in HIV (Human Immunodeficiency Virus) care [30], maternal health [31], and tuberculosis monitoring [32,33].

Participants preferred a mixed-interface model combining checkboxes, dropdowns, and limited open-text fields to reduce data entry time, an approach shown to improve usability in India and Kenya [34,35]. Offline capability was considered essential, reflecting connectivity challenges observed in Malawi and Tanzania [36]. While multi-component apps may be complex, phased introduction and ongoing training, as used in SMARTHealth [37], were viewed as acceptable. Interoperability with DHIS2 was a universal requirement, consistent with global recommendations for scalable digital health systems [38].

DHT members stressed the need for device provision, stable internet, and power backups. Integration with DHIS2 and the inclusion of dementia-specific indicators would reduce duplication and improve data quality [39,40]. Infrastructure limitations seen in South Africa and other LMICs [7,41,42] can be mitigated through offline functionality and hybrid synchronization models [43]. Embedding performance indicators and real-time dashboards, as used in mTrac and RapidSMS [44,45], was seen as vital for supervision and accountability.

To reduce redundancy, eCap-CoDe should align with existing HMIS workflows and expand to include other common conditions, a strategy that improved adoption in OpenSRP and Medic Mobile [46]. Self-paced training within the app addresses persistent knowledge gaps [47,48], while real-time feedback loops can enhance diagnostic accuracy and care quality [49–51]. Offline data capture and simplified entry processes are non-negotiable for sustaining use [36,52], alongside localized training, mentorship, and supervision [53,54].

### Implications for Uganda and the global context

In Uganda, eCap-CoDe directly addresses PHC system gaps by combining in-app dementia training, structured data capture, and real-time feedback with seamless integration into HMIS/DHIS2. Embedding dementia indicators into national reporting could improve surveillance, planning, and resource allocation for mental health, aligning with the country’s digital health transformation agenda [55]. Design features such as offline functionality and user-friendly workflows address persistent rural infrastructure challenges [31].

For national scale-up, policymakers should:

1. Integrate mHealth governance into Uganda’s eHealth strategy, ensuring interoperability and alignment with WHO Digital Health Guidelines [1].
2. Establish financing models such as public–private partnerships, donor support, to sustain deployment [56].
3. Incorporate task-shifting policies enabling CHWs to use mHealth tools effectively.
4. Enforce data security and privacy safeguards that balance global standards with local realities [43].

Globally, this study adds to the sparse literature on co-designed, provider-facing digital dementia tools in LMICs, complementing the WHO mhGAP-IG and advancing the WHO Global Action Plan on Dementia 2017–2025. eCap-CoDe’s integrated approach, comprising training, service delivery support, and actionable feedback, provides a scalable and adaptable model for addressing other mental and neurological disorders in similar contexts, thereby supporting data-driven decision-making, enhancing care quality, and promoting health system resilience.

### Ethics statement

The researchers recognize that rural settings have varying levels of digital literacy, which can influence the ethical implementation of digital health interventions. In designing and implementing the eCap-CoDe intervention, we prioritized informed consent, secure data storage, access control, and user training to ensure patient confidentiality and data security. Given the potential vulnerability of sensitive health information, we will adhere to the General Data Protection Regulation (GDPR) and the Health Insurance Portability and Accountability Act (HIPAA) to safeguard patient data [57–59]. To mitigate risks of unauthorized access, particularly in areas with intermittent connectivity, we will implement encrypted data transmission, role-based access control, and offline-first data collection mechanisms [43]. Drawing on lessons from the offline-first app [56], the intervention will incorporate educational initiatives to train PHC providers on data privacy best practices while ensuring culturally appropriate consent processes that accommodate varying digital literacy levels. Furthermore, we recommend regular audits and compliance checks to identify and address vulnerabilities, in line with global best practices for upholding ethical data-handling standards in digital health systems operating in resource-limited settings [60].

## Strengths and limitations

This study is among the first in Uganda to explore the perspectives of both PHC providers and DHT members on the design requirements for a mobile health application to support dementia care. Using a co-design approach and the Consolidated Framework for Implementation Research, the findings reflect real-world workflows, organizational processes, and frontline priorities, enhancing the potential for acceptability, uptake, and sustainability. Participant diversity across cadres and facilities strengthened the relevance of insights for both provider- and system-level needs.

However, the focus on two rural districts may limit generalizability to urban or peri-urban contexts, and reliance on self-reported data introduces the potential for social desirability bias. As a formative qualitative study conducted prior to development and pilot testing, findings do not capture real-world user interactions or technical performance.

## Conclusion

Overall, the results provide a practical blueprint for the development of context-appropriate digital tools for dementia care in rural LMIC settings. Pilot testing of eCap-CoDe is now essential to assess feasibility, usability, and potential impact, and to inform iterative refinement of the application prior to scale-up within national health information systems.

## Supporting information

S1 File Interview guide for Primary Health Care (PHC) providers and District Health Team (DHT) members. Semi-structured interview guide used to explore perceived barriers and facilitators to dementia care and digital tool use, and to inform the co-design of the eCap-CoDe mobile application. The guide includes questions on content requirements, user experience, organizational considerations, and service delivery needs, informed by the Consolidated Framework for Implementation Research (CFIR).
(DOCX)
