# Supplementary material for: Co-designing eCap-CoDe: A mobile health application for primary health care-based dementia care in rural Uganda
Source: PLOS Digit Health. 2026 Apr 30;5(4):e0001389. doi: 10.1371/journal.pdig.0001389 (PMC13132442; doi:10.1371/journal.pdig.0001389)
Supplement: S1 File — Semi-structured interview guide used to explore perceived barriers and facilitators to dementia care and digital tool use, and to inform the co-design of the eCap-CoDe mobile application. The guide includes questions on content requirements, user experience, organizational considerations, and service delivery needs, informed by the Consolidated Framework for Implementation Research (CFIR). (DOCX) [file pdig.0001389.s001.docx]

## **Interview guide for PHC providers**

**Title:** A multi-component mHealth intervention ‘eCap-CoDe’ consisting of training, data capture, and feedback tailored for improved community-based dementia care in rural Uganda: the barriers, ideating, and development process

**Introduction**

The purpose of this study is to identify barriers and facilitators faced by PHC providers as it relates to training, data collection, and feedback on community-based assessment and management of dementia, and to co-design a multi-component intervention, eCap-CoDe, to improve community-based dementia care in rural Uganda. I will ask you about content and user experience requirements when designing a mobile application. There is no right or wrong answer. Please answer each question in detail. Your truthful answers will help develop a multi-component intervention of training, data collection, and feedback to improve community-based dementia care.

**Socio-demographics**

| 1. Age 2. Sex 3. Level of Education 4. Marital status | 1. Employment /health cadre 2. Years of employment in the position 3. District of employment |
| --- | --- |

| **CFIR domain** | **Requirements** | **Exemplar questions to ask** |
| --- | --- | --- |
| Individual | Content requirement | *Training needs*   1. Tell me about the availability older adults ≥60 years at this facility. Approximately how many do you see in a month? 2. What screening tools do you use to screen behavioral or cognitive functions of older adults ≥60 years? What challenges do you have with dementia assessment and management? 3. If the challenges were addressed, how committed are you to performing dementia assessment and management? 4. What kind of support do you provide to caregivers of dementia patients?   *Data capture [dementia]*   1. How do you record data on dementia assessment and management? 2. What challenges do you face with the process of data collection? 3. How does the data you collect get to the HMIS?   *Feedback*   1. Who provides feedback for improvement after data is compiled from the HMIS registers? 2. How do you receive feedback on dementia assessment and management? 3. Who should give feedback to the PHC providers on dementia assessment and management? |
| Process |  | 1. What technical support will you need to use ‘eCap-CoDe’ mobile application? *Probes: training, data capture, and feedback* 2. What do you think the role of LHWs is in linking older adults ≥60 years to healthcare to promote utilization of the ‘eCap-CoDe’ intervention? 3. How best should the LHWs support the process of in linking the older adults ≥60 years to healthcare? 4. What do you think about continuous evaluation and improvement during implementation of the ‘eCap-CoDe’ intervention? 5. How do you think that it should be done to best help you improve? 6. How often should the continuous evaluation be done? |
| Inner setting |  | 1. How would your work structure influence your ability to implement the proposed ‘eCap-CoDe’ intervention? 2. How would electronic data analysis, visualization, storage, and reporting generation support performance of the health facility? 3. How would utilization of the ‘eCap-CoDe’ intervention influence relational connections with your peers at the health facility? |
| Innovation characteristics | User experience Requirements | 1. What do you think about the proposed multi-component intervention ‘eCap-CoDe’? *Probe for complexity, adaptability, cost* 2. What features would you want to see included in the mobile application? 3. How friendly do you think the interface should be? *Probe preference for checkboxes, dropdown, textbox* 4. How can ‘eCap-CoDe’ be integrated with the existing workflows to ensure seamless data exchange? 5. What advantage do you think that ‘eCap-CoDe’ has over the other interventions that you have used? |

## **Interview Guide District Health Team**

**Title:** A multi-component mHealth intervention ‘eCap-CoDe’ consisting of training, data capture, and feedback tailored for improved community-based dementia care in rural Uganda: the barriers, ideating, and development process

**Introduction**

The purpose of this study is to identify barriers and facilitators faced by PHC providers as it relates to training, data collection, and feedback on community-based assessment and management of dementia, and to co-design a multi-component intervention, eCap-CoDe, to improve community-based dementia care in rural Uganda. I will ask you a few questions about your role as a healthcare authority at the district level. There is no right or wrong answer. Please answer each question in detail. Your truthful answers will help develop a multi-component intervention of training, data collection, and feedback to improve community-based dementia care.

**Socio-demographics**

| 1. Age 2. Sex 3. Level of Education 4. Marital status | | | 1. Employment /health cadre 2. Years of employment in the position 3. District of employment |  |
| --- | --- | --- | --- | --- |
| **CFIR domain** | **Requirements** | **Exemplar questions to ask** | | |
| Outer setting | Organizational requirement | 1. What are your thoughts about a multi-component mobile application for PHC providers consisting of (a) training on dementia assessment and management, (b) data capture of the diagnosis and management through the phone application linked to the HMIS platform, and (c) feedback to the PHC providers on the diagnosis and management for improvement? 2. What organizational systems or workflows should be considered when designing the mobile App to enable seamless data capture and feedback? 3. What performance measures need to be included in the application to help you track and encourage implementation by the PHC providers? 4. What support will you need to supervise the PHC providers ably and provide them with data-informed feedback? | | |
|  | Service requirements | 1. How do you feel about ‘eCap-CoDe’ and its integration with the HMIS? 2. What modifications need to be made to the current health management information system to accommodate or successfully consolidate the data that will potentially be collected using the mobile phone application? 3. How would you support the PHC providers in utilizing the multi-component intervention? 4. What kind of reports should the HMIS team expect from ‘eCap-CoDe’ intervention-generated data? | | |
